# Effects of a *Saccharomyces cerevisiae* fermentation product on liver abscesses, fecal microbiome, and resistome in feedlot cattle raised without antibiotics

**DOI:** 10.1038/s41598-019-39181-7

**Published:** 2019-02-22

**Authors:** Katherine L. Huebner, Jennifer N. Martin, Carla J. Weissend, Katlyn L. Holzer, Jennifer K. Parker, Steven M. Lakin, Enrique Doster, Margaret D. Weinroth, Zaid Abdo, Dale R. Woerner, Jessica L. Metcalf, Ifigenia Geornaras, Tony C. Bryant, Paul S. Morley, Keith E. Belk

**Affiliations:** 1 Department of Clinical Sciences, Colorado State University, Fort Collins, Colorado, USA; 2 Department of Animal Sciences, Colorado State University, Fort Collins, Colorado, USA; 3 Department of Microbiology, Immunology, and Pathology, Colorado State University, Fort Collins, Colorado, USA; 4 Five Rivers Cattle Feeding, LLC, Kersey, Colorado, USA

## Abstract

Liver abscesses in feedlot cattle form secondary to high concentrate feeds and rumen acidosis. Antimicrobial drugs are commonly included in cattle feed for prevention of liver abscesses, but concerns regarding antimicrobial resistance have increased the need for alternative treatments. A block randomized clinical trial was conducted to evaluate the effects of a *Saccharomyces cerevisiae* fermentation product (SCFP) on liver abscesses, fecal microbiomes, and resistomes in cattle raised without antibiotics in a Colorado feedlot. At enrollment, steers (n = 4,689) were sorted, by weight and source, into 2 pens comprising a block (n = 14 blocks, 28 pens); pens were randomly allocated to either the control group or the treatment group, where the diet was supplemented with SCFP. Prior to harvest, composited feces were collected for characterization of the microbiome and resistome using 16S rRNA gene and shotgun sequencing. At harvest, liver abscess severity was quantified for individual cattle. There were no statistical differences detected by treatment group in animal health, liver abscess prevalence or severity. Organisms classified to phylum, Elusimicrobia were more abundant in the feces of treated cattle, however, there were no differences in the resistome by treatment group. Both microbiome and resistome varied significantly among enrollment blocks.

## Introduction

The occurrence of liver abscesses is a common problem in cattle that is associated with feeding diets containing highly fermentable carbohydrates (i.e., starch), among other factors, during the finishing period. These high grain diets are the primary underlying cause of clinical and subclinical ruminal acidosis, rumenitis, bacterial translocation into the portal circulation, and subsequent liver abscess formation^1,2^. Consequently, there are detrimental impacts for animal production and welfare, and the diminished value of animals at slaughter result in significant economic loss to the beef cattle industry^3–6^. Results of the recent National Beef Quality Audit-2016 demonstrated that the occurrence of liver abscesses in feedlot cattle has increased^7^. Traditionally, management practices used to control and prevent liver abscessation include nutritional management and inclusion of antibiotics in the feed or water. In the U.S., tylosin phosphate (a macrolide antibiotic) and chlortetracycline are approved for inclusion in cattle diets for the control of liver abscesses, and over 70% of cattle in large commercial beef feedlots have been reported to receive tylosin in diets for this purpose^8^.

Antimicrobial drugs effectively control liver abscesses, but the impact of antimicrobial exposures on antimicrobial resistance (AMR) is currently of great concern to public health. Antimicrobial resistance in cattle has been traditionally evaluated using culture-based approaches^9,10^, but the impacts of antimicrobial drug treatments to the microbial communities (the microbiome) or on the entire portfolio of AMR genes (the resistome) have only recently started to be explored^11^. Due to growing scrutiny regarding the use of medically important antimicrobials in livestock production, the use of tylosin as a feed additive for growth promotion in livestock was banned in the European Union; in the United States, the use of tylosin for this purpose has recently been placed under veterinary oversight with the Veterinary Feed Directive. Future regulatory requirements may restrict the duration for use of tylosin for this purpose in livestock, which has implications for animal health, productivity, and welfare. Thus, reducing the use of antimicrobial drugs and development of alternative treatment strategies are critical areas for feedlot cattle production and an active area of research. These products will be particularly important in treating feedlot cattle that have higher susceptibility to the formation liver abscesses, such as beef breeds raised without antibiotics (‘natural cattle’) and steers originating from dairy farms that are raised for beef production^1^.

Several non-antimicrobial products and feed additives have been evaluated for their effectiveness in controlling subacute rumen acidosis and liver abscesses, including sunflower seeds^12,13^, essential oils^14,15^, and vaccines^16,17^. *Saccharomyces cerevisiae* fermentation products (SCFP) have also been evaluated for use as feed additives in dairy and beef production. Some studies have shown that SCFP can positively impact gastrointestinal health for cattle exposed to high-concentrate diets, by providing components including amino acids, peptides, organic acids, and oligosaccharides^18–20^. In some studies, inclusion of SCFP in cattle diets improved performance characteristics, including increased milk production^21^, increased volatile fatty acid concentration, decreased rumen pH^22^, efficient feed conversion, and improved carcass traits^23^. Other studies regarding SCFP supplementation reported reductions in AMR and food safety pathogens detected in the feces, as well as lymph node carriage of virulent *Salmonella enterica*^24^. A previous clinical trial found no significant difference in the prevalence of liver abscesses in feedlot cattle fed diets containing SCFP in comparison to cattle fed diets containing tylosin, suggesting there may be similar effectiveness for the control of liver abscesses in cattle^25^.

We hypothesized that dietary supplementation with SCFP could impact the occurrence of liver abscesses, cattle health and performance, microbial communities, and resistomes through modulation of subacute rumen acidosis in feedlot cattle exposed to a high concentrate diet. A block randomized clinical trial was performed to evaluate these outcomes in the feces of beef cattle raised without exposure to antimicrobial drugs or growth-promoting hormones in a commercial feedlot. The outcomes evaluated in this clinical trial in response to SCFP supplementation included the following: the prevalence and severity of liver abscesses, animal health outcomes and feedlot performance, the fecal microbiome (using 16S rRNA sequencing), and the fecal resistome, including genes related to antimicrobial, metal, and biocide resistance (using shotgun metagenomic sequencing).

## Results

### Animal health, production performance, and liver abscess prevalence

In total, 4,689 steers were randomly enrolled in the study; 2,345 steers housed in 14 pens were administered the control diet, and 2,344 cattle housed in 14 pens were administered a diet supplemented with SCFP. The number of cattle housed in the 28 pens used for this study ranged from 104 to 225 animals per pen (average = 167 cattle). Overall, 278 steers required antimicrobial treatment for disease in compliance with veterinary protocols, and 41 cattle died during the study, including both steers removed from the pen for treatment in addition to cattle found dead within the pen; these animals were removed from further analysis in the trial. Controlling for hierarchical population structure, there were no detectable differences in production (average daily gain (ADG) and dry matter intake (DMI) to gain ratio) or animal health variables (crude or cause-specific morbidity and mortality) between treatment groups receiving the diet containing SCFP or the control ration (Tables 1 and 2; *P* > 0.05). Across all pens, the duration of days on feed (DOF) ranged from 171 to 262 d, with an average of 205 d; there were no significant differences between SCFP treatment and control groups (*P* = 0.81).

**Table 1:** Baseline and production performance data summary, averaged at the pen-level.

Experimental Group^a^				
Variable^b^	Control (n = 28)	SCFP Treatment (n = 28)	Standard Error^c^	*P* value
Days on feed	203.90	205.60	6.99	0.81
Daily dry matter intake (lbs/animal/day)	22.21	22.21	0.32	0.99
Average daily gain (lbs/animal/day)	2.90	2.87	0.06	0.74
Dry matter intake:gain	7.69	7.75	0.13	0.65

^a^Refer to text for description of the treatment protocols.

^b^Refer to text for pen level production variable calculations.

^c^Calculated using standard generalized linear model of pen averages for experimental group effects and correcting for intra-pen clustering within block.

**Table 2:** Animal health data summary^a^.

Experimental Group^b^	Control (n = 2345)			SCFP Treatment (n = 2344)			
Animal Health Variable	Adjusted risk (%)	95% CI^c^	n	Adjusted risk (%)	95% CI^c^	n	*P* value
Crude Morbidity	7.2	5.4–9.6	168.0	8.3	6.0–11.3	194.0	0.51
Respiratory	4.6	3.4–6.4	109.0	5.7	3.8–8.5	134.0	0.44
Digestive	0.0	N/A	0.0	0.0	N/A	0.0	N/A
Other Causes	0.9	0.4–1.9	21.0	0.6	0.2–1.5	14.0	0.51
Crude Mortality	0.7	0.5–1.2	17.0	1.0	0.8–1.3	24.0	0.17
Respiratory	N/A^d^	N/A^d^	0.0	N/A^d^	N/A^d^	4.0	N/A^d^
Digestive	0.3	0.1–0.5	7.0	0.5	0.3–0.7	11.0	0.14
Other Causes	N/A^d^	N/A^d^	10.0	N/A^d^	N/A^d^	9.0	N/A^d^

^a^Refer to text for animal health variable calculations and protocols for handling of sick or treated animals.

^b^Refer to text for description of the treatment protocols.

^c^Calculated for each adjusted rate using Poisson regression in a log linear model for experimental group effects and correcting for intra-pen clustering within block with generalized estimating equations.

^d^Not applicable (NA) denotes where the model did not converge, due to the small number of events.

In total, the livers from 4,324 individual cattle were evaluated at slaughter to assess the presence and severity of liver abscesses using the standardized scoring system. Controlling for randomization block, the adjusted risk for cattle having a detectable liver abscess, of any severity score, was 38.9% (95% CI = 38.0 to 44.5) and 38.1% (95% CI = 31.4 to 46.2) for cattle fed the control ration or cattle fed the SCFP ration, respectively (Table 3). The difference in abscess risk was not statistically significant between treatment groups (*P* = 0.79). Similarly, there were no statistically detectable differences between treatment groups in the risk for abscesses of different severity, i.e., severe (A-plus), moderate (A) or minor (A-minus); (*P* > 0.05).

**Table 3:** Liver abscess prevalence, by grade and treatment group.

Experimental Group^a^	Control (n = 2171)			SCFP Treatment (n = 2153)			
Liver Abscess Score^b^	Adjusted risk (%)^c^	95% CI	n	Adjusted risk (%)	95% CI	n	*P* value
A-minus	17.6	15.0–20.6	382.0	17.4	15.0–20.2	375.0	0.93
A	5.7	3.9–8.5	122.0	5.1	3.3–7.8	106.0	0.26
A-plus	15.2	14.1–16.5	341.0	15.3	14.1–16.5	337.0	0.98
Total abscesses	38.9	34.0–44.5	845.0	38.1	31.4–46.2	818.0	0.79

^a^Refer to text for description of the treatment protocols.

^b^Refer to text for standardized scoring system for gross liver pathology at slaughter.

^c^Adjusted risk was calculated for each adjusted rate using Poisson regression in a log linear model for treatment group effects and correcting for pen clustering within block with generalized estimating equations.

### Fecal microbiome composition and diversity

High quality 16S rRNA amplicon sequences totaling 13,749,784 reads were obtained from the 28 composited fecal samples and imported into Qiime2-2017.12 for fecal microbiome characterization^26^; the median Phred quality score for forward and reverse reads was 38 to 39, respectively (Supplementary Fig. S1). Using the DADA2 pipeline^27^ and filtering out chloroplasts of plant origin and mitochondrial sequences, 85% of reads passed quality control. Read count summaries, by sample and processing step, are shown in Supplementary Table S1.

**Figure 1:**
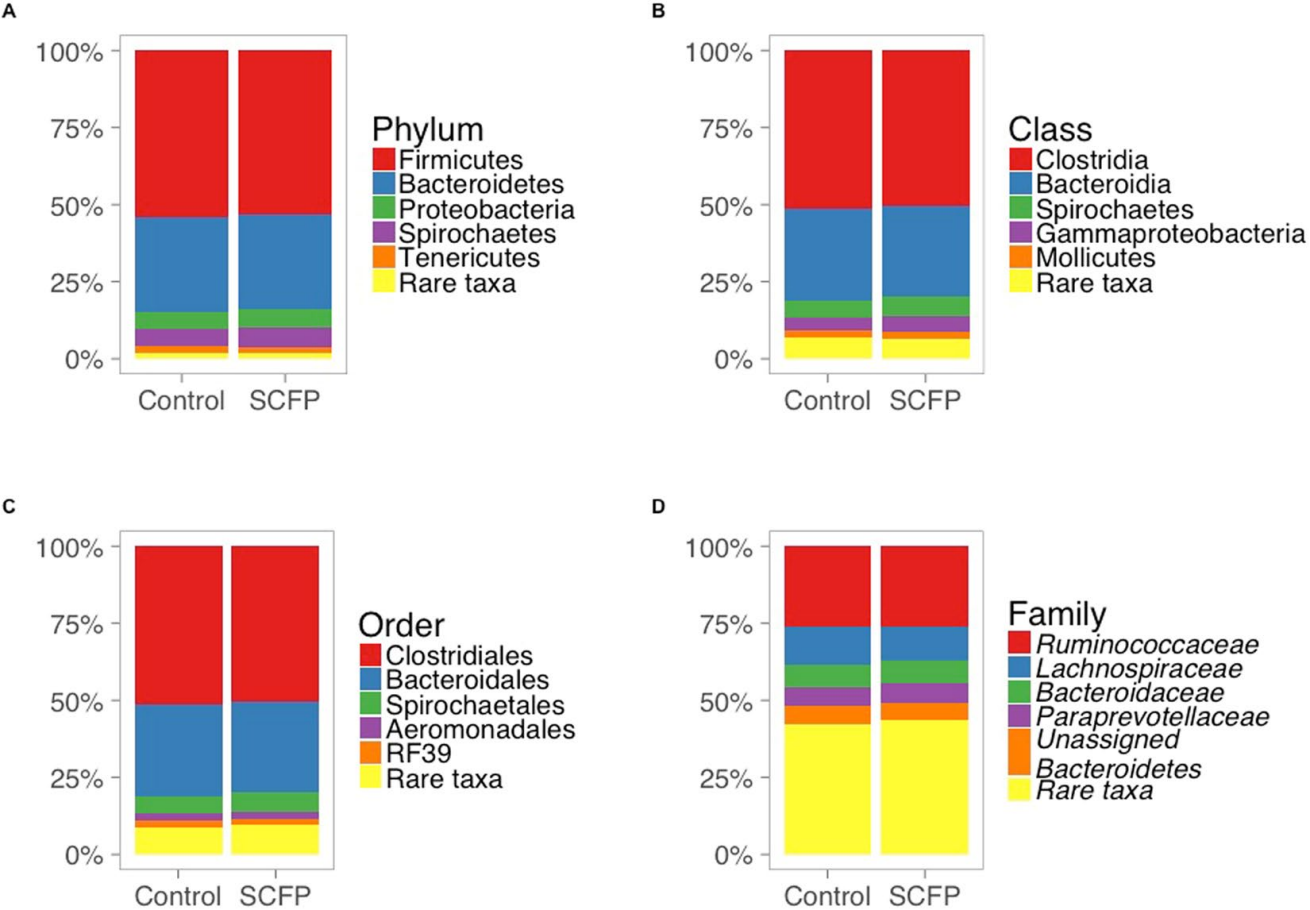
Percent relative abundance (count within taxonomic group/total count × 100) of composited fecal microbiome at phylum, class, order, and family-levels across 28 samples collected from cattle supplemented with SCFP or the control diet. The top five abundant taxonomic classifications for sequence variants are labelled, and rare taxa (defined as having a percent relative abundance <2%) were summed and put into a distinct category, “rare taxa”, at each level.

The fecal microbiome of composite samples collected from both treatment groups across 28 samples was dominated by the phyla Firmicutes, Bacteroidetes, Proteobacteria, Spirochaetes, and Tenericutes (Fig. 1), and at the family level Ruminococcaceae, Lachnospiraceae, Bacteroidaceae, and Paraprevotellaceae were the predominant taxonomic features in both treatment groups. Additionally, there were 46 rare phyla which accounted for <1% of all reads across 28 samples (Supplementary Table S2). Taxa within the rare phyla category included Actinobacteria, Acidobacter, Fibrobacteres, Verrucomicrobia, Cyanobacteria, Nitrospirae, Chloroflexi, Gemmatimonadetes, Planctomycetes, Euryarchaeota (kingdom Archaea), WS3, Elusimicrobia, Fusobacteria, TM7, WPS-2, Armatimonadetes, Chlamydiae, Chlorobi, AD3, OP3, TM6, Lentisphaerae, Synergistes, FCPU426, GN04, WWE1, Deferribacteres, BRC1, NC10, FBP, Caldithrix, Thermi, OD1, Crenarchaeota (kingdom Archaea), GOUTA4, NKB19, GN02, SC4, OP8, BHI80-139, SBR1093, LCP-89 and OC31.

Adjusted estimates of abundance revealed that 1 SV classification to the phylum, Elusimicrobia, was present in significantly higher abundance within the composited fecal microbiome from SCFP-fed pens of cattle compared to cattle fed the control ration. However, while Elusimicrobia was detected in 100% of the composited fecal samples, this pattern of higher abundance in SCFP treated cattle was not observed in 4 of 14 blocks. Though the differences were not considered biologically significant due to low relative abundance, SVs that classified to the phylum, Fusobacteria, were present in higher relative abundance within the fecal microbiome of cattle fed the control ration; however, this effect was only present within 3 of 14 blocks. No other SVs were differentially abundant between treatment groups, at any taxonomic level (Supplementary Table S3).

**Figure 2:**
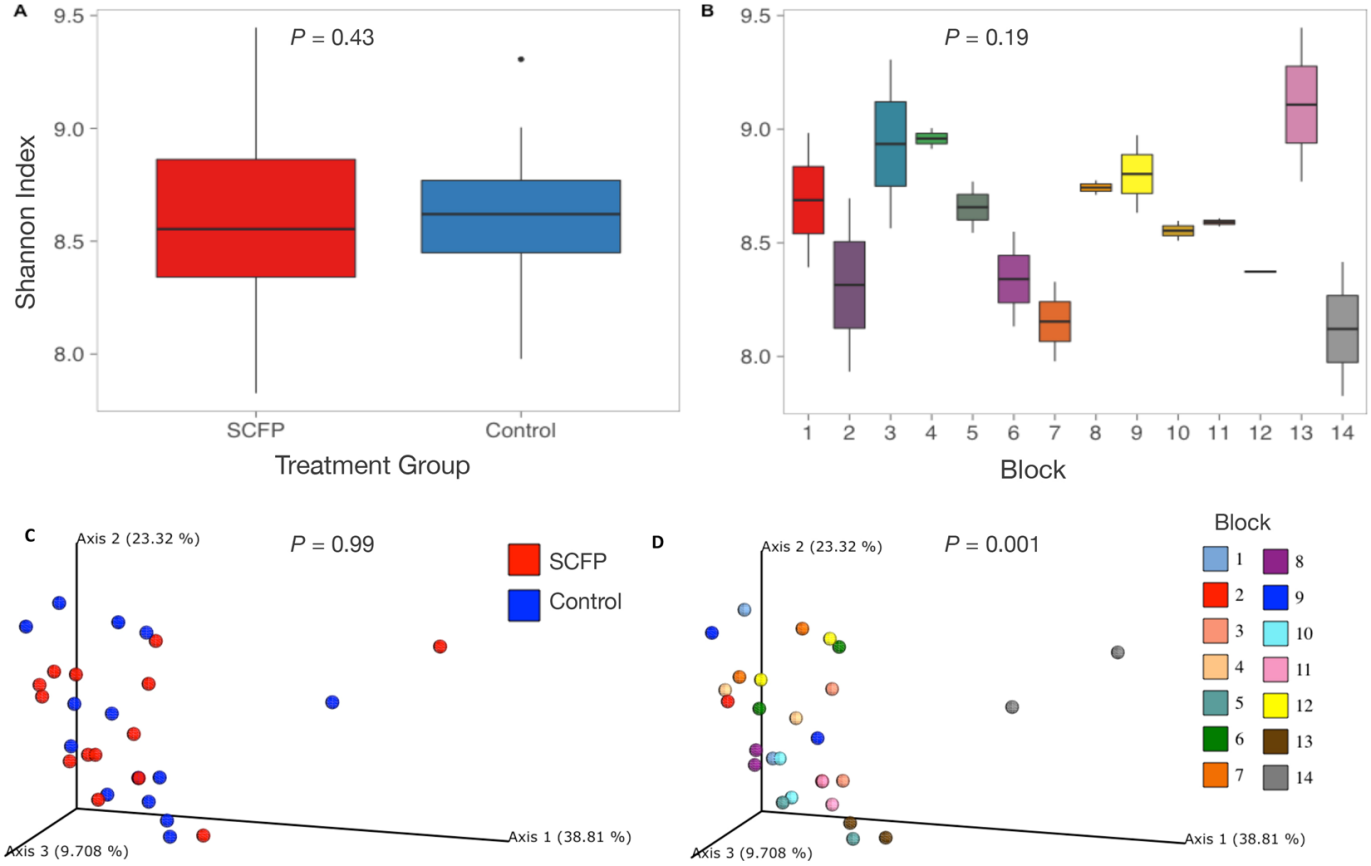
Diversity metrics for the composited fecal microbiome, by treatment group and enrollment group (block). Shannon alpha diversity, or within-sample microbial diversity, did not differ by treatment group (**A**) or block (**B**). Statistical differences for alpha diversity were determined using non-parametric Kruskall-Wallis tests. Beta diversity, or microbial community composition, visualized as principal coordinates analysis ordination based on 16S rRNA gene sequence weighted UniFrac distance, differed by treatment group (**C**) but did not differ by block (**D**).

Microbial diversity, estimated using the Shannon diversity index on rarefied counts, did not differ between the SCFP supplemented and control pens of cattle (Fig. 2A; *P* = 0.43). Though not significant, Shannon alpha diversity varied among blocks, with lower diversity in block 14 (Fig. 2B; *P* = 0.19). Degree of differentiation in the microbial communities, or beta diversity, was not significantly different by treatment group (Fig. 2C; *P* = 0.99); however, block-related effects accounted for the significant differences in the composited fecal microbiome (Fig. 2D; *P* = 0.001). The first axis explained 38.8% of the variation, which was largely driven by differences in the microbiome of the composite fecal sample collected from pens in block 14.

### Resistome composition and diversity

Shotgun sequencing generated 994,459,164 pairs of forward and reverse reads from 28 composite fecal samples. The average quality score across samples was 38 (range 36 to 38), indicating an expected error rate of 1.6 out of 10,000 base calls from sequencing (Supplementary Table S4). Following quality filtering and removal of reads that aligned to the *Bos taurus* genome, 895,733,367 read pairs remained for downstream analysis. Of these, 1,610,843 read pairs mapped to the gene accessions in the MEGARes and BacMet databases for elements encoding for antimicrobial drug and biocide resistance, respectively. There were no significant differences in the number of raw or trimmed reads or quality scores between the treatment and control groups, suggesting that there was no systematic bias in the sequencing effort between treatment groups.

Overall, 364,482 read alignments aligned to the MEGARes database (i.e., “hits”), and were classified to 35 mechanisms for antibiotic drug, metal, and biocide resistance. Single nucleotide polymorphisms were confirmed as described in the methods for housekeeping genes that confer resistance. At the mechanism level, 0.27% of hits mapped to determinants for metal or biocide resistance and 99.73% of reads mapped to determinants for resistance to antimicrobial drugs (Supplementary Table S5). At the mechanism level, most read alignments were to AMR determinants for tetracycline resistance ribosomal protection proteins (62.3%) and macrolide resistance efflux pumps (25.6%).

Reads aligning to determinants for ribosomal protection protein mechanisms were the most abundant hits within the tetracycline resistance class, including *tetQ*, *tetO*, *tetW*, *tet32, tet44, tet40*, and *tetW*. Other hits within the tetracycline drug class included inactivation enzymes (*tetX*) and the major facilitator superfamily (MFS) efflux pumps (*tetA*, *tetB*). Reads aligning to determinants for macrolide efflux pumps (*MefA*) comprised the majority of hits to the macrolide, lincosamide, and streptogramin resistance class, followed by 23S rRNA methyltransferase target modification genes (*ermq*), macrolide phosphotransferase genes (*mphb*), and the lincosamide nucleotidyltransferases (*lunc*). For hits to determinants in the aminoglycoside class, aminoglycoside O-nucleotidyltransferase mechanisms (*ant9*) were most abundant. Within the beta-lactam class, alignments to the class A beta-lactamase (*cfx*) AMR mechanisms were abundant.

Among reads aligning to AMR determinants for biocides, biocide resistance proteins (*glpF*) and biocide resistance regulators (*rpoS, gadA* and *sugE*) were the most abundant. Of alignments that mapped to AMR determinants for metal resistant mechanisms, most of them conferred resistance to multiple metals.

**Figure 3:**
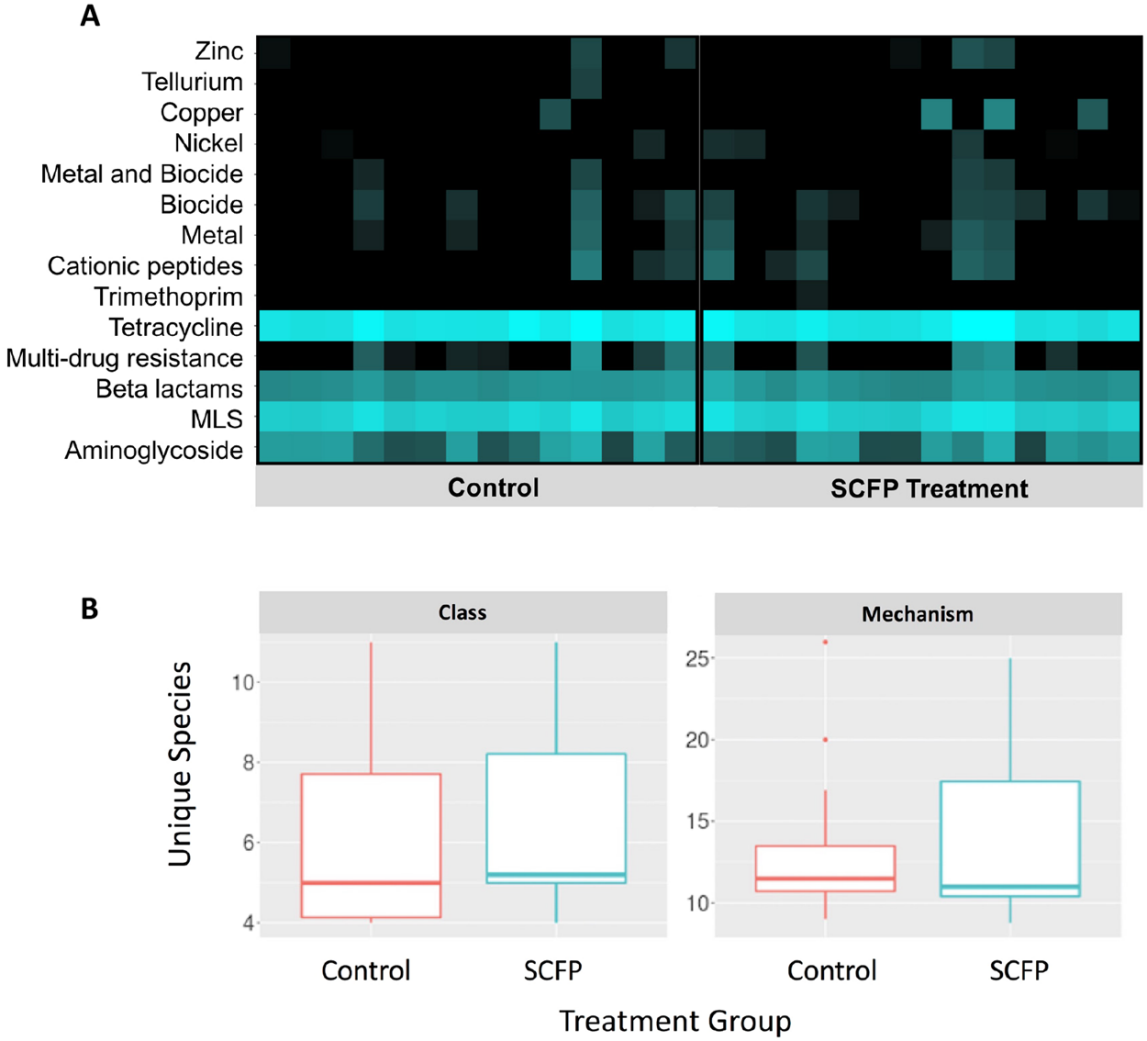
Heatmap showing treatment comparison of the normalized counts of antimicrobial, metal and biocide resistance determinants in the composited fecal resistome by drug class. Each column represents one composited fecal sample, grouped by treatment (**A**). Boxplot species richness comparison for composited fecal resistome at the class and mechanism levels, measured as unique species using inverse Simpson’s index, by treatment group (**B**).

**Figure 4:**
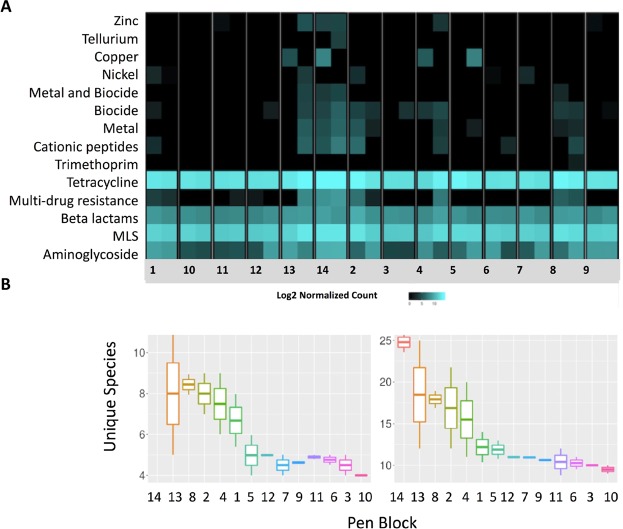
Heatmap showing block comparison of normalized counts of antimicrobial, metal and biocide resistance genes, by drug class. Each column represents one composited fecal sample, by enrollment group (block) (**A**). Boxplot species richness comparison for fecal resistome at the class and mechanism level measured as unique species measured by inverse Simpson’s index, by block (**B**).

The composition and taxonomic richness of antimicrobial, metal and biocide resistance determinants did not differ significantly between treatment groups at expression levels high enough (the cut-off value was > 1) to infer biological significance at any taxonomic level (Fig. 3A,B). Likewise, NMDS ordination did not show significant separation between the 2 treatment groups, at any taxonomic level. However, the resistome composition and richness differed when evaluating the resistome changes among blocks (Fig. 4A,B), and this shift was observed at the Mechanism and Class levels (average expression levels >1, *P* < 0.001).

For critically important AMR resistance gene accessions that were specifically screened, only 1 sample collected from a pen of cattle supplemented with SCFP was found to contain alignments to an extended spectrum beta-lactamase (*ctx)* determinant. However, there were only 23 reads aligning to this determinant (0.038% of the total alignments for AMR determinants in that sample).

## Discussion

Results of this block randomized clinical trial showed that supplementation with SCFP did not significantly affect liver abscess prevalence, the fecal microbiome, or the resistome in commercial feedlot cattle raised without antimicrobial drug exposures. There were also no detectable treatment differences in animal health or performance outcomes. Overall, this study population expressed a high prevalence of liver abscesses (38%), which is approximately twice the national average for commercial beef cattle within the United States^7^. The relatively high liver abscess prevalence in this trial was likely attributable to the absence of antimicrobial inclusion in the trial diets, whereas previous reports regarding liver abscess prevalence were estimated for cattle populations in which tylosin was administered in the feed. This high liver abscess prevalence may have impacted the ability to detect effectiveness of SCFP as a control measure. For example, in a a previous study that demonstrated reduced (although insignificant) numbers of liver abscesses in cattle fed SCFP compared to a diet including tylosin and ionophores, there were overall lower prevalence for both total and severe liver abscesses (10–20%)^25^.

The results of this study identified significant variation across randomization block with respect to the liver abscess prevalence, fecal microbiome and fecal resistome. It is possible that this significant variation by block may have obscured subtle treatment effects of SCFP supplementation. As cattle were enrolled into the trial, they were randomly sorted into adjacent pens until a randomization block was filled, then randomly assigned to receive either the treatment or control diet. This design ensured that treatment groups of cattle within each block had equivalent distribution of a variety of factors, including cattle origin (i.e., rearing source), weight, pen location, and season of placement. The blocks were intended to control for potential confounding factors, such that they were evenly distributed between treatment groups to reduce the likelihood that differences related to these variable factors would bias the study outcome. However, the significant differences among blocks suggested that these factors could be strong drivers of microbial ecology and resistomes in cattle feces, and potentially liver abscess prevalence. These factors, including cattle source, rearing environments, and influences from within the feedlot may have a greater impact on the microbiome and liver abscess occurrence than has been previously understood, and warrant consideration in the design of future dietary feeding trials and studies designed to understand risk factors for liver abscess occurrence.

Our evaluation of composited feces obtained from the cattle prior to slaughter suggested that overall, there were only minor differences between treatment groups. Only 1 SV classifying to the phylum, Elusimicrobia, had a higher relative abundance in the feces of SCFP supplemented groups compared to the control group. There is limited information in the literature regarding Elusimicrobia in feedlot cattle, however, it has been associated in cattle diagnosed with periodontal disease^28^. There were no other significant treatment effects detected regarding the resistome or other outcomes measured in this clinical trial, which may be due to several reasons, including the randomization block variation, SCFP dose, sample type, or sample numbers.

While the SCFP was supplemented at the manufacturer’s recommended dosage, the daily exposure dose of SCFP that was used in this clinical trial may have been too low to result in detectable differences in the fecal microbiome or resistome. One study reported linear increases in cellulolytic and lactate-utilizing bacteria associated with increasing concentrations of SCFP in the diet^29^. However, that study evaluated rumen, not fecal samples, and the dose of SCFP was up to 10 times higher than the levels of SCFP fed in this trial.

Feed additives containing SCFP have been hypothesized to modulate rumen fermentation, yet this trial only characterized fecal composited samples. This limitation was primarily due to several practical limitations associated with the handling of a large populations of owned commercial feedlot cattle. Therefore, the conclusions from this trial regarding impact of SCFP supplementation are restricted to impacts on the fecal microbiome and resistome. The gastrointestinal microbiome of cattle has been previously characterized as heterogeneous with respect to microbiome diversity and composition across distinct anatomical compartments^30,31^. However, some studies have demonstrated that cattle exposed to grain-induced, subacute rumen acidosis challenges have shifts in both the fecal microbial composition as well as in the rumen^30^. Interestingly, metagenomic characterizations of the microbiome and resistome of feedlot cattle following exposures to feed additives (tylosin and monensin) have been shown to have minimal to no impact on the in rumen, cecum, and small intestine contents^11^. Furthermore, epimural rumen microbial communities (bacteria adherent to the rumen epithelial lining) may have greater impact on liver abscess pathogenesis as compared to microbial populations found in liquid or solid fractions of rumen contents^2,32,33^, and investigation of these microbiome niche communities are warranted. Future work directed towards evaluation of alternative feed additives, including SCFP, would benefit from additional evaluations of other gut compartments, including the rumen and rumen epimural microbial communities, in addition to investigations using fecal samples.

The predominant AMR classes detected within the feces of cattle were tetracycline resistance ribosomal protection proteins, macrolide resistant efflux pumps, beta-lactamases, and aminoglycoside resistance. This resistome profile is consistent with other published metagenomics studies of feedlot and dairy cattle^34–37^. The cattle enrolled in this study were not treated with any antimicrobial drugs within their lifetime, and the pens used to house these cattle were only used to manage cattle raised without antimicrobial drug exposures, so cross-contamination with treated populations were unlikely. As such, it is interesting that several AMR mechanisms were detected even though this population of cattle did not have previous antimicrobial exposures. This is highlighted by the identification of small numbers of reads aligning to extended spectrum, beta-lactamase gene accessions (*ctx*), belonging to the Ambler class A beta-lactamases group, and further highlights the complexity of ecologies related to AMR in feedlot operations and other agricultural production settings. The *ctx* gene confers resistance to third-generation cephalosporins (e.g., cefotaxime), and is classified as critically important threat to human health when expressed in disease-causing agents, such as *Escherichia coli*^38,39^. This AMR gene was previously identified in another study of feedlot cattle that characterized the fecal resistome in populations raised without antibiotics, although the abundance was higher in the conventionally-treated cattle comparison group^34^.

Despite several advantages, metagenomics approaches are not without limitation. For example, there is a lack of established methods for performing accurate power calculations in metagenomics studies designed for applied research in commercial feedlot settings. This is partly due to lack of baseline information about cattle microbiomes and resistomes exposed to diverse management conditions, including different antimicrobial treatment exposures. As a result, it may be possible that the effects of SCFP treatment may have been obscured by the significant variation in microbiomes across block, driven by one of several potentially confounded factors. Future studies could be designed to evaluate cattle exposed to feed additives through use of more replicates at the pen-level, through use of individuals as the experimental unit, or through collection of other sample types. Additionally, future work should be directed towards investigation of underlying factors that influence the microbiome, independent of dietary treatment groups, which could influence impact the underlying risk factors for formation and severity of liver abscesses in feedlot cattle.

## Materials and Methods

### Trial design and study population

**Figure 5:**
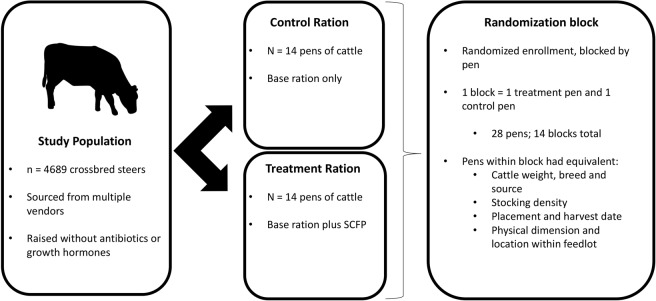
Overview of the block randomized study design. Crossbred steers were purchased in groups and transported to a commercial feedlot in Northern Colorado. Upon arrival, animals from each vendor were processed separately. Cattle were individually weighed using a chute, and heavy or light weight animals (relative to the weight distribution for the source population) were excluded. Remaining cattle were randomly allocated to the 2 treatment groups, which were housed in separated, adjacent pens. Randomized enrollment was blocked by pen, so that cattle were allocated to a pair of pens within a block. Pens within a block had equivalent distributions for cattle weights, breeds, sources, stocking density, and placement and harvest dates. The pens had equivalent dimension, and animals were housed in open-air, dirt-floor pens with a central feed alley. Pens of cattle within each randomization block were managed using identical protocols for cattle movements, timing of feed delivery, feed bunk management, and daily health inspection. Cattle housed together in a pen were shipped for harvest based on standard procedures to determine when they have reached market weight (1400–1500 lbs). Treatment and control pens within each block were shipped for slaughter on the same day, in the same order that blocks were randomly enrolled into the trial.

Methods involving animal care and use were approved by and carried out in accordance with the CSU Research Integrity and Compliance Review Office (Protocol number 102616). In a block randomized clinical trial study design, cattle were allocated to a pen that received either a treatment or control diet; each treatment and control pair made up one randomization block (Fig. 5). The study population was comprised of cattle that were approximately 50% purebred Angus, and crossbreds between Angus and English or continental breeds (i.e., no known Brahman or dairy influence). Yearling steers were purchased directly from domestic producers located across multiple western states during March through June 2016. Eligible cattle were reared through their lives without exposure to antimicrobial drugs or growth promoters to enable marketing of natural beef products. At arrival, cattle were individually weighed and administered injectable and oral anti-parasitic treatments (Noromectin; Synanthic; Standguard), injectable bacterin-toxoid vaccines protecting against respiratory disease complex agents (Titanium-5 + PH-M), and clostridial diseases (Vision-7). The pens used within the feedlot to house these cattle were only used to manage cattle raised without antimicrobial drug exposures.

Except for SCFP supplementation included in the diet of treated cattle, the diets fed to both treatment groups were identical, and were formulated to meet the National Research Council nutritional requirements^40^. The SCFP product (NaturSafe, Diamond V) was supplemented to treated cattle diets at the manufacturer’s recommended dose of 17.8 g/d (Supplementary Table S6). Cattle started receiving the treatment diets when they were transitioned to the finishing ration starting week 2 to 3 following placements. Cattle were fed twice per day. Separate equipment was used for preparation and delivery of treatment diets, and the mill and trucks were sanitized between diets to minimize cross-contamination.

Trained personnel, masked to treatment assignment, evaluated cattle for signs of illness at arrival and daily under the supervision of a veterinarian. When identified, ill or injured animals were moved for examination, and primary illness was classified by body system (i.e., respiratory, digestive, or other diseases). Cattle requiring treatment with antibiotics, as per trial protocols, were moved and permanently housed elsewhere. Cattle that died underwent a necropsy by a veterinarian for cause of death classification. The cattle were harvested between October to December 2016.

### Sample and Data collection

One day prior to harvest, a composited sample of fresh feces was collected from the pen floor for each pen within a block (n = 28 fecal samples), using previously described methods^37,41^. Briefly, composites were created by combining several samples of freshly voided feces (~10 g each) taken using a gloved hand from the top and center of individual fecal pats from 20 locations along crossing diagonals of each pen of cattle. The fecal composites were transported on ice within insulated containers within 1 h of collection to the laboratory at Colorado State University for further processing (described below). Animal health and weight data were collected, and individual animal weights were summarized through calculation of arithmetic mean, by pen. The ADG was calculated using the difference of pen-averaged slaughter weight and starting weight divided by the DOF. The DMI to gain ratio was calculated by dividing daily DMI of feed by ADG. Crude morbidity attack rate was calculated as the number of sick animals, whether they received treatment or not, divided by the initial number of animals assigned to each pen. Crude mortality attack rate was calculated as the number of animals that died, whether they received treatment or not, divided by the initial total number of animals assigned to each pen. Cause-specific morbidity attack rates were calculated as the number animals treated and divided by the initial number of animals in the pen. Cause-specific mortality attack rates for disease categories were calculated as the number of animals that died, whether they died in the pen or after removal for antimicrobial treatment, divided by the total number of animals assigned to each the pen.

The cattle were euthanized using approved procedures at a commercial abattoir. Identities of feedlot pens (not treatment group assignments as all personnel remained masked to treatment group throughout the study) were tracked in the slaughter plant to allow for determination of liver abscess occurrence by pen and treatment group. After evisceration, the presence and severity of liver abscesses for individual animals were classified by a trained observer using a modified Elanco Liver Check System, as previously described^6^. Briefly, the external surfaces of livers were observed and palpated. Livers without visible abscesses were scored as normal, and livers were classified as A-minus, A or A-plus to denote increasing severity of abscessation. Other pathological liver abnormalities were not scored.

### Sample processing and sequencing

Within the collection bags, fecal composites were manually homogenized for 1 minute each, and aliquots were weighed and stored at −80 °C. DNA was isolated from samples using commercial kits (PowerMax Soil DNA Isolation Kit) with modifications as previously described^42^. Briefly, 10 g of sample aliquots were thawed at 4 °C, and a sedimentation step was performed to remove large particulates and sediment layers. The resulting pellet was resuspended in PowerBead solution, and the remainder of the standard DNA extraction kit protocol followed. DNA concentration and quality were evaluated using a NanoDrop^TM^ spectrophotometer (Thermo Fisher Scientific, Inc.). Samples with 260:280 nm ratios >1.8 and DNA concentrations >20 ng/μl were sent for sequencing; samples that did not meet the concentration threshold were concentrated by ethanol precipitation before sequencing.

The DNA quantification results for negative control samples were evaluated following extraction with only kit reagents (blank extractions). Sample concentrations and DNA quantification results following PCR amplification were assessed using agarose gel electrophoresis of the genomic DNA and PCR products following amplification. The PCR products from blank samples extracted during this study’s sample extractions were obtained only after several rounds of amplification and were of insufficient quantity for sequencing.

The 16S rRNA gene amplification and sequencing for composited fecal sample DNA was performed by a commercial sequencing company (Novogene Corporation). Following isolation, DNA aliquots (1500 ng, 30 µl) were delivered to the sequencing facility. DNA from the V4 region of the 16S rRNA gene was amplified using the primer set 515F/806R^43^, with reverse primers containing unique barcodes. Library sequencing (paired-end, 2 × 250 bp) was performed on a HiSeq. 2500 Sequencing System (Illumina).

Fecal samples were also sequenced using shotgun sequencing. DNA aliquots (3 µg, 50 µl) were delivered to the University of Colorado Genomics and Microarray Core for sequencing. Genomic libraries for all samples were prepared using the TruSeq DNA PCR-Free Library Prep Kit (Illumina) following the manufacturer’s protocol to obtain an average insert size of 350 bp. Library sequencing was completed on the HiSeq 4000 Sequencing System (Illumina) with 17 samples loaded into each lane, v4 chemistry and paired-end reads of 150 nucleotides in length.

### Bioinformatics and statistical analysis

Treatment group assignments were masked during data analysis. The 16S rRNA gene forward and reverse reads were imported into Qiime2-2017.12^26^. The DADA2 pipeline^27^ was used for detecting and correcting Illumina amplicon sequences, removal of primers and chimeric reads, and assembly into sequence variants (SV). Quality filtering included primer trimming and sequence truncation to remove low quality sequences. Taxonomy was assigned using a naïve Bayes classifier^44^ trained on the Greengenes 13_8_99% database. Sequences classified as chloroplasts, mitochondria, and sequences with a count less than 10 were filtered. A rooted phylogenetic tree was created through *de novo* multiple sequence alignment using MAFFT, vs.7^45^, highly variable positions were removed to decrease noise in the tree, and FastTree-2 was applied to generate a tree from the masked alignment^46^. The feature table was rarefied to 65,000 sequences, allowing retention of all samples. Beta diversity analysis using the distance matrix generated from the weighted UniFrac phylogenetic metric^42^ was visualized in a principle coordinates analysis (PCoA), using Emperor^47^. Alpha diversity was estimated using the Shannon diversity metric.

The SV count data were extracted from Qiime2, and imported with metadata into the “metagenomeSeq” R package (v1.20.1)^48^. Taxonomic lineage for each SV was identified and alignments summarized by phylum, class, order, and family. The count data were normalized using cumulative sum scaling (using default percentile of 0.5 for normalization) and analyzed using zero-inflated Gaussian mixed-model regression, with treatment as a fixed effect and block as a random effect. Log-fold changes and *P*-values for regression coefficients were calculated by calculating pair-wise contrasts between treatment levels using Limma’s “makeContrasts” function^49^. *P*-values were corrected for multiple testing using the Benjamini-Hochberg false discovery rate^50^. Testing for significant effects of categorical metadata, including treatment group, for alpha and beta diversity metrics were performed with rarefied feature tables (as described above).

The AmrPlusPlus pipeline v.1.20.0^51^ was used to characterize AMR at the class, mechanism and group levels from the shotgun metagenomics data. The pipeline was executed using Nextflow (v.0.26.0)^52^. The sequence data were filtered and quality controlled using Trimmomatic (v0.36)^53^. Host (bovine) genomic DNA contamination was removed by aligning the quality controlled reads to the *Bos taurus* genome (NCBI accession AC_000158.1) using the Burrows Wheeler Aligner (BWA-MEM)^54^, then removing the *B. taurus* genome using SAMtools^55^. Remaining reads were aligned to the MEGARes AMR resistance database (v1.01) and hand-annotated genes from the BacMet database using BWA-MEM^56^. An 80% gene fraction threshold (i.e., 80% of the full length of each AMR gene accession within each sample) was applied to identify potential positive AMR gene accessions.

The count data for AMR determinants were normalized using cumulative sum scaling and analyzed using zero-inflated Gaussian mixed-model regression in the “metagenomeSeq” R package (v1.20.1)^48^, with treatment as a fixed effect and block as a random effect. Measures for alpha diversity were calculated on data normalized to the lowest sample size using the vegan package (v.2.4-6)^57^. Ordination was performed using non-metric multidimensional scaling (NMDS) with Bray-Curtis distance. Log-fold changes, expression levels, and *P*-values for regression coefficients were calculated with pair-wise contrasts between treatment levels. *P*-values were corrected for multiple testing using the Benjamini-Hochberg false discovery rate^50^. Differential abundance testing for AMR mechanism and class were only considered biologically significant at expression levels >1. As previously described^37^, the resistome from composited fecal samples in this study was specifically screened for selected high profile resistance genes and drugs of concern to public health.

All animal health and performance were analyzed using SAS, (SAS Institute, release 9.4) using pen as the experimental unit. Animal health variables were compared between treatment groups using mixed-effects Poisson regression, with block specified as a random effect. Performance variables were compared between treatment groups in a generalized linear model adjusting for pen and block structures. Least squares means were significantly different if *P* was ≤0.05.

## Supplementary information

SUPPLEMENTAL TABLES AND FIGURES

Table S3

Table S6

## Data Availability

Quality-trimmed, concatenated sequencing reads for the 28 shotgun metagenomic sequencing reads described have been uploaded to the NCBI collection of biological data (BioProject) in Accession PRJNA453374 ID 453374. Raw, paired end reads for the 28 16S rRNA gene V4 region sequence sets have been uploaded to the NCBI collection of biological data (BioProject) in Accession PRJNA472376 ID 4058025.
