# Supplementary material for: Effects of a Saccharomyces cerevisiae fermentation product on liver abscesses, fecal microbiome, and resistome in feedlot cattle raised without antibiotics
Source: Sci Rep. 2019 Feb 22;9:2559. doi: 10.1038/s41598-019-39181-7 (PMC6385275; doi:10.1038/s41598-019-39181-7)
Supplement: Supplementary file 1 — SUPPLEMENTAL TABLES AND FIGURES [file 41598_2019_39181_MOESM1_ESM.pdf]

SUPPLEMENTAL TABLES AND FIGURES

Effects of a *Saccharomyces cerevisiae* fermentation product on liver abscesses, fecal microbiome, and resistome in feedlot cattle raised without antibiotics

Katherine L. Huebner,[a*] Jennifer N. Martin,[b] Carla J. Weissend,[b] Katlyn L. Holzer,[b] Jennifer K. Parker,[a] Steven M. Lakin,[a] Enrique Doster,[c] Margaret D. Weinroth,[b] Zaid Abdo,[c] Dale R. Woerner,[b] Jessica L. Metcalf,[b] Ifigenia Geornaras,[b] Tony C. Bryant,[d] Paul S. Morley,[ac] Keith E. Belk[b#]

[a]Department of Clinical Sciences, Colorado State University, Fort Collins, Colorado, USA

[b]Department of Animal Sciences, Colorado State University, Fort Collins, Colorado, USA

[c]Department of Microbiology, Immunology, and Pathology, Colorado State University, Fort Collins, Colorado, USA

[d]Five Rivers Cattle Feeding, LLC, Kersey, Colorado, USA

Running head: Saccharomyces supplementation to prevent liver abscesses

#Address correspondence to Keith E. Belk, Keith.Belk@colostate.edu.

*Present address: Katherine L. Huebner, Center for Veterinary Medicine, Food and Drug Administration, Rockville, Maryland.

P.S.M. and K.E.B. contributed equally to this work.

Table S1: 16S rRNA amplicon reads by frequencies and summary statistics at different stages of data processing, including raw fastq sequences, following quality control in the DADA2 pipeline, and following filtering out mitochondrial and chloroplast sequences.

Sample ID	Imported Sequences	Following DADA2	Following Filtering
**CF1**	405942	348298	348298
**CF2**	436851	372814	372814
**CF3**	643540	520266	519923
**CF4**	465200	380085	380085
**CF5**	540561	467300	467300
**CF6**	1002314	852917	852917
**CF7**	327779	279321	279321
**CF8**	871911	744616	744613
**CF9**	600316	511071	511056
**CF10**	430094	359935	359677
**CF11**	366452	316713	316713
**CF12**	248945	218523	218518
**CF13**	277280	228743	228640
**CF14**	468365	401412	401406
**CF15**	696914	595602	595602
**CF16**	489723	410888	410888
**CF17**	282386	240601	240501
**CF18**	485072	415209	415209
**CF19**	352641	294369	294369
**CF20**	544153	466501	466501
**CF21**	504813	429127	429127
**CF22**	244950	207346	207346
**CF23**	451262	382182	382182
**CF24**	456146	388754	388754
**CF25**	482651	404829	404829
**CF26**	785434	650689	650684
**CF27**	505038	376912	376912
**CF28**	383051	307061	307056
**Minimum frequency**	244,950	207,346	207,346
**1st quartile**	378,901	314,300	314,299
**3rd quartile**	541,459	466,701	466,701
**Maximum frequency**	1,002,314	852,917	852,917
**Total reads**	13,749,784	413,289	413,259

Table S2: Sequence variants (SV) raw relative counts and proportions classified at phyla level in the fecal composite microbiome, by treatment group. There was a high abundance of rare SV, defined as SV accounting proportionally for < 2 % of all classified SV counts across 28 samples.

	**Control**		**SCFP Treatment**	
**Sequence variant classified at Phylum level**	**Total count**	**Proportion of total (%)**	**Total count**	**Proportion of total (%)**
Firmicutes	2886139	54.01346	3297505	52.94755
Bacteroidetes	1639273	30.67864	1915458	30.75622
Proteobacteria	305045	5.70885	395594	6.35199
Spirochaetes	287367	5.37801	379433	6.09250
Tenericutes	131877	2.46805	137689	2.21085
Actinobacteria	40875	0.76497	36542	0.58675
Acidobacteria	12991	0.24312	20966	0.33665
Fibrobacteres	12460	0.23319	7264	0.11664
Unassigned bacteria 1	5461	0.10220	7054	0.11327
Verrucomicrobia	4341	0.08124	6888	0.11060
Cyanobacteria	4097	0.07667	6422	0.10312
Nitrospirae	2083	0.03898	2883	0.04629
Chloroflexi	2158	0.04039	2760	0.04432
Gemmatimonadetes	2199	0.04115	2557	0.04106
Planctomycetes	1596	0.02987	2349	0.03772
Euryarchaeota (Kingdom Archaea)	934	0.01748	1378	0.02213
WS3	692	0.01295	1035	0.01662
Elusimicrobia	421	0.00788	812	0.01304
Unassigned bacteria 2	530	0.00992	626	0.01005
Fusobacteria	817	0.01529	218	0.00350
TM7	309	0.00578	571	0.00917
WPS-2	436	0.00816	160	0.00257
Armatimonadetes	322	0.00603	245	0.00393
Chlamydiae	172	0.00322	348	0.00559
Chlorobi	160	0.00299	291	0.00467
AD3	78	0.00146	166	0.00267
OP3	104	0.00195	78	0.00125
TM6	61	0.00114	119	0.00191
Lentisphaerae	92	0.00172	85	0.00136

Synergistetes	68	0.00127	99	0.00159
FCPU426	32	0.00060	77	0.00124
GN04	62	0.00116	16	0.00026
WWE1	6	0.00011	45	0.00072
Deferribacteres	4	0.00007	32	0.00051
BRC1	19	0.00036	12	0.00019
NC10	8	0.00015	18	0.00029
FBP	20	0.00037	5	0.00008
[Caldithrix]	11	0.00021	12	0.00019
[Thermi]	15	0.00028	8	0.00013
OD1	5	0.00009	14	0.00022
Crenarchaeota (Kingdom Archaea	4	0.00007	11	0.00018
GOUTA4	9	0.00017	0	0.00000
NKB19	9	0.00017	0	0.00000
GN02	0	0.00000	8	0.00013
SC4	0	0.00000	8	0.00013
OP8	0	0.00000	5	0.00008
BHI80-139	3	0.00006	0	0.00000
SBR1093	3	0.00006	0	0.00000
WS2	0	0.00000	3	0.00005
LCP-89	0	0.00000	2	0.00003
OC31	2	0.00004	0	0.00000

Table S4: Shotgun sequencing paired-end reads frequencies and summary statistics at different stages of data processing. Raw fastq sequences, after quality control and host removal, and number of alignments to the BacMet and MEGARes databases using the AmrPlusPlus pipeline.

Sample	Paired-end reads/sample)	Quality Score	After quality control host removal	Read alignments to database
CF1	43209371	37.7	39299378	67839
CF2	23216028	37.7	20907069	36996
CF3	24793292	37.7	22067343	33189
CF4	14853690	38.1	13419546	20511
CF5	23619704	37.9	21248030	43974
CF6	32284007	37.8	29014215	54721
CF7	19068839	36.1	17070809	28006
CF8	25819778	38.2	23502170	40019
CF9	56382659	37.7	51280927	93491
CF10	26320094	37.6	23648974	47166
CF11	23994450	37.8	21604334	38358
CF12	35921580	38.5	31916136	61311
CF13	24403110	37.8	22075567	40913
CF14	36985682	37.6	33248902	61155
CF15	50191940	36.5	44936754	76617
CF16	56687773	38.5	50853345	83789
CF17	27444715	37.7	24551659	35608
CF18	48122715	37.7	43544670	82627
CF19	40797472	36.4	37006659	57137
CF20	33062421	38.4	30127464	51521
CF21	26471970	37.9	23538950	38710
CF22	25310627	37.7	22951381	36447
CF23	47525379	37.8	43246811	95286
CF24	43772475	38.4	39254620	70683
CF25	56028423	38.0	50543553	95303
CF26	47536926	37.9	42552740	80368
CF27	50357870	37.9	45186233	89420
CF28	30276174	38.3	27135128	49678
Minimum	14853690.0	36.1	13419546.0	20511.0
Quartile 1	25181293.3	37.7	22732427.5	38622.0
Mean	35516398.7	37.8	31990477.4	57530.1
Quartile 3	47528265.8	38.0	42726257.8	77554.8
Maximum	56687773.0	38.5	51280927.0	95303.0

Table S5: Number and proportion of reads aligning to each category and mechanism of resistance.

Resistance Mechanism	Total reads aligned	Proportion of reads
**Antimicrobial Drug Resistance**		
Tetracycline resistance ribosomal protection proteins	226,940	62.264%
Tetracycline inactivation enzymes	1,285	0.353%
Macrolide phosphotransferases	3,672	1.007%
Tetracycline resistance major facilitator superfamily MFS efflux pumps	4,212	1.156%
Aminoglycoside O-phosphotransferases	195	0.054%
Aminoglycoside N-acetyltransferases	26	0.007%
Penicillin binding protein	132	0.036%
Class C betalactamases	29	0.008%
Lincosamide nucleotidyltransferases	9,820	2.694%
Aminoglycoside efflux pumps	299	0.082%
Macrolide resistance efflux pumps	93,254	25.585%
Aminoglycoside efflux regulator	24	0.007%
Aminoglycoside O-nucleotidyltransferases	9,130	2.505%
Polymyxin B resistance regulator	90	0.025%
Multi-drug efflux pumps	1,031	0.283%
23S rRNA methyltransferases	1,786	0.490%
Dihydrofolate reductase	2	0.001%
Class A betalactamases	10,855	2.978%
MDR regulator	455	0.125%
Lipid A modification	273	0.075%

Resistance Mechanism	Total reads aligned	Proportion of reads
**Biocide Resistance**		
Biocide resistance protein	135	0.037%
Biocide resistance regulator	31	0.009%
**Metal Resistance**		
Metal efflux pump	13	0.004%
Metal resistance protein	87	0.024%
Metal efflux regulator	57	0.016%
Metal efflux protein	8	0.002%
Metal ABC efflux pump	13	0.004%
Nickel ABC efflux pump	18	0.005%
Nickel ABC efflux regulator	6	0.002%
Copper resistance protein	465	0.128%
Tellurium resistance protein	13	0.004%
Zinc ABC efflux pump	7	0.002%
Zinc resistance regulator	64	0.018%
Metal resistance regulator	9	0.002%
**Cross-Category Resistance**		
Metal and biocide resistance protein	46	0.013%
**Total**		
Total Reads	364,482	

Table S6: Dry matter ingredient and chemical composition for basal finishing diets averaged over the feeding period.

**Ingredient**	**DM Inclusion, % (St.Dev)**
Steam-flaked corn	63.1
Corn silage	12.6
Grass hay	1.9
Corn stalks	0.2
WDG	5.1
DDG[a]	8.7
Whey delactose permeate	1.8
Supplement	4.0
Vegetable oil	2.6
Feed additive[b]	
Chemical Composition	
Crude Protein, %	14.7 (0.79)
NDF, %	16.2 (1.58)
Ca, %	0.5 (0.06)
P, %	0.4 (0.04)

[a]The *Saccharomyces cerevisiae* fermentation product (SCFP; NaturSafe, Diamond V, Cedar Rapids, IA) treatment diet contained a separate supplement at 3.2% (dry matter basis (DM) comprised of Dried Distillers grains (DDG) as the carrier and SCFP; this supplement displaced DDG in the basal diet.

[b]SCFP was supplemented at the manufacturer's recommended dose of 17.8 g/d (vs. target of 18.0 g/d; 1,603 g/ton DM basis) for the treatment group.

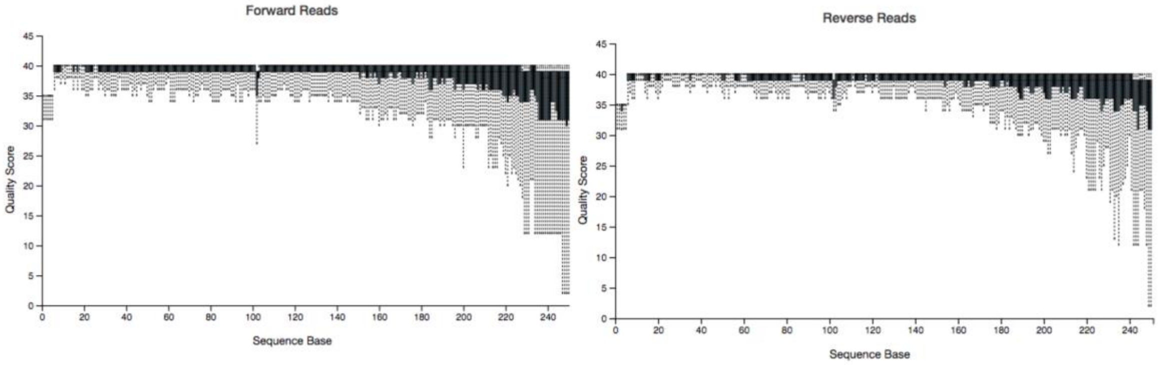

Figure S1: Summary of quality scores (i.e., Phred Score) by sequence base position in the forward and reverse raw reads generated through 16S rRNA gene sequencing.
